# Two Quenchers Formed During Photodamage of Phostosystem II and The Role of One Quencher in Preemptive Photoprotection

**DOI:** 10.1038/s41598-019-53030-7

**Published:** 2019-11-21

**Authors:** Alonso Zavafer, Ievgeniia Iermak, Mun Hon Cheah, Wah Soon Chow

**Affiliations:** 1 Research School of Biology, College of Science, The Australian National University, Canberra, ACT 2601 Australia; 2 Laboratory of Biophysics, Wageningen University, P.O. Box 8128, 6700 ET Wageningen, The Netherlands; 3 BioSolar Cells Project Office, P.O. Box 98, 6700 AB Wageningen, The Netherlands; 4 Present Address: São Carlos Institute of Physics, University of São Paulo, São Carlos, SP Brazil; 5 Present Address: Department of Chemistry, Uppsala University, Uppsala, Sweden

**Keywords:** Fluorescence imaging, Bioenergetics, Light responses, Photosystem II, Photobiology

## Abstract

The quenching of chlorophyll fluorescence caused by photodamage of Photosystem II (qI) is a well recognized phenomenon, where the nature and physiological role of which are still debatable. Paradoxically, photodamage to the reaction centre of Photosystem II is supposed to be alleviated by excitation quenching mechanisms which manifest as fluorescence quenchers. Here we investigated the time course of PSII photodamage *in vivo* and *in vitro* and that of picosecond time-resolved chlorophyll fluorescence (quencher formation). Two long-lived fluorescence quenching processes during photodamage were observed and were formed at different speeds. The slow-developing quenching process exhibited a time course similar to that of the accumulation of photodamaged PSII, while the fast-developing process took place faster than the light-induced PSII damage. We attribute the slow process to the accumulation of photodamaged PSII and the fast process to an independent quenching mechanism that precedes PSII photodamage and that alleviates the inactivation of the PSII reaction centre.

## Introduction

Photosynthesis is the biochemical reaction that sustains most ecosystems on Earth. However, this process has an intrinsic suicidal nature, as the light absorbed by the photosynthetic machinery leads to chemical modifications (photodamage) causing its inactivation (photoinactivation)^1^. Among all the components of photosynthesis, photosystem II (PSII) is most vulnerable to photodamage^2^. Two types of photoinactivation can be identified^3,4^: Total PSII and PSII reaction centre (RC) photoinactivation. Total PSII photoinactivation refers to light-induced loss of the oxygen evolution (a process that requires an active Mn_4_CaO_5_ cluster and an active RC). On the other hand, RC photoinactivation involves inhibition of the capacity of RC to reduce Q_A_, the primary quinone acceptor of electrons. While there is no consensus about the causes, primary site(s) and molecular mechanism of total PSII photodamage^1,5^, it is accepted that RC photodamage is mostly dependent on excitation pressure and driven by pigment absorption.

Based on photosensitisers, two hypotheses have been proposed to explain total PSII photodamage. The *light energy absorption by photosynthetic pigments model* proposes that photodamage is caused by excessive photon absorption or/and charge recombination reactions^5–11^. By contrast, the *Mn photoinactivation model* suggests that photodamage of PSII is caused by direct absorption of light by the Mn_4_CaO_5_ cluster^3,12^ within PSII and is independent of excessive excitation of chlorophyll.

It is widely accepted that PSII photodamage is alleviated by protection mechanisms, collectively termed photoprotection^13^. As the main cause of photodamage has been considered to be excessive absorption of energy, non-photochemical quenching (NPQ), has been proposed as a way to dissipate the excessive excitation that can otherwise induce PSII photodamage^13,14^. For supporters of the Mn photoinactivation model, the physiological role of NPQ is not to avoid Total PSII damage but to protect RC and the PSII repair mechanisms^15^. Even though most reports about NPQ acknowledge its role as a mechanism of Total PSII photoprotection^13,14,16–18^, experimental evidence suggests that quenching of excitation has low efficiency in protecting against the light-induced loss of the Total PSII activity^15,19–21^. Thorough studies that analyse the relationship between NPQ and photoprotection have been reported^22^, but most studies do not take into consideration either wavelength dependence of photodamage or inhibition of PSII repair.

One of the consequences of PSII inactivation is fluorescence quenching. This type of quenching, termed qI, has been attributed to several causes and primarily to long-lasting qE and qZ mechanisms (such as ΔpH, xanthophylls, etc). However, it was demonstrated, by measuring chlorophyll fluorescence lifetime, that even in the absence of NPQ mechanisms (particularly qE and qZ) quenching still occurs^23^. The origin and physiological role of qI mechanisms remain unknown.

In order to gain further insights into the qI, we investigated the time course of the decrease of average fluorescence lifetime (τ_AV_) to scrutinize its role in photodamage. To see if the decrease of τ_AV_ only occurs in leaves or is intrinsic to PSII, changes in τ_AV_ were evaluated during the time course of PSII photodamage *in vitro*. Two experimental model systems under the excessive excitation were compared: photorepair-impaired spinach leaves (*in vivo* system) and PSII enriched membranes (here on referred to as BBY’s, an *in vitro* system). Since the decrease in τ_AV_ might be related to damage of the Mn_4_CaO_5_ cluster or induced by photosynthetic pigments, we compared the effect of blue light (where more damage to Mn_4_CaO_5_ cluster has been observed) versus red light where photodamage is mostly driven by photosynthetic pigments. The time-dependent PSII activity decrease and quenching fluorescence lifetime are compared to the time course of PSII photoinactivation.

## Results

### PSII photodamage is accompanied by shortening of τ_AV_

**Figure 1:**
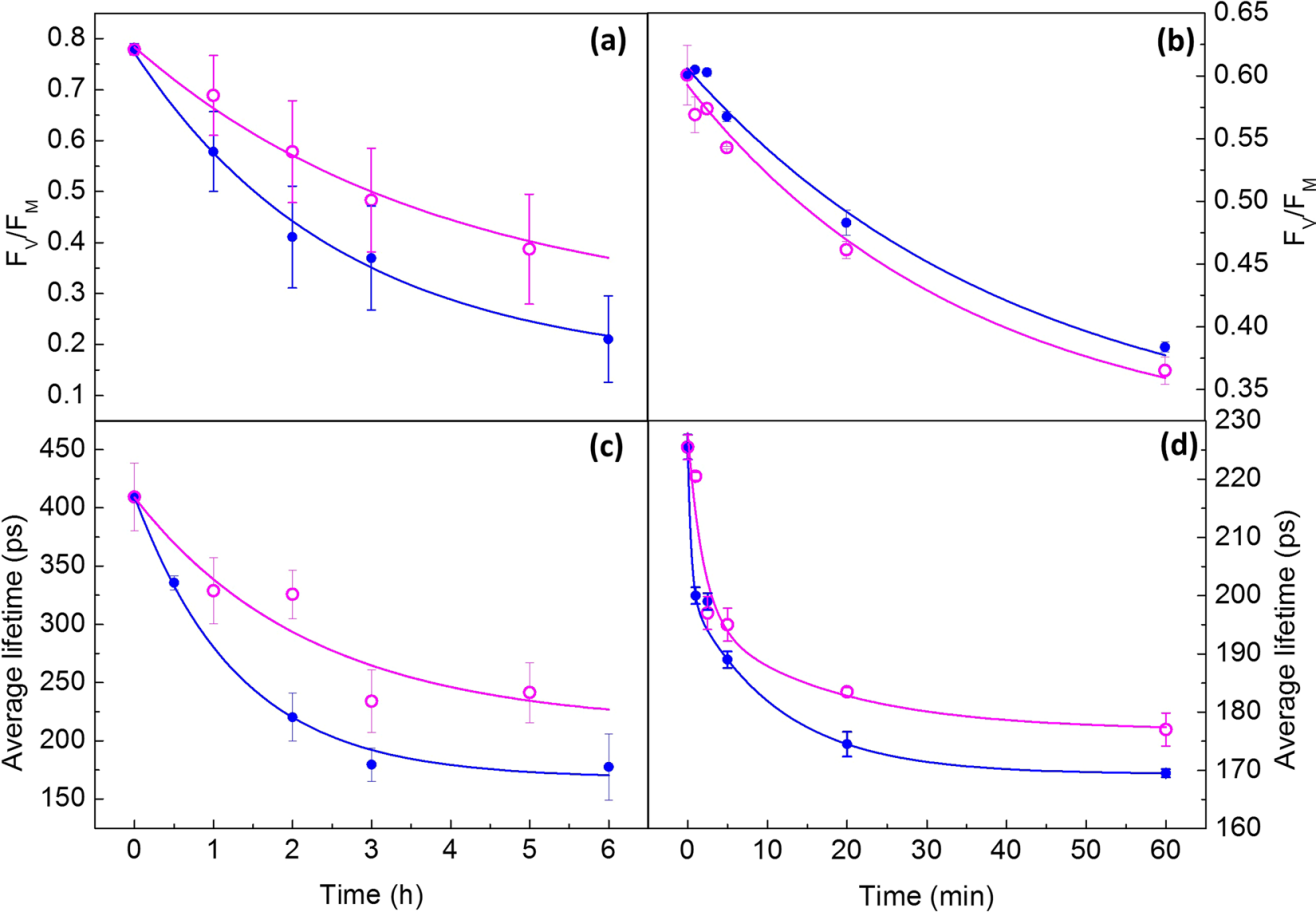
Changes in PSII efficiency and average fluorescence lifetimes after illumination with 460 nm (blue data points) and 660 nm (purple data points) light. Effect of photodamage on PSII efficiency (F_V_/F_M_) in (**a**) spinach leaves, (**b**) BBY’s. Changes in average fluorescence lifetimes upon illumination, measured on (**c**) spinach leaves, (**d**) BBY’s. Fitting results using Eqs 2 and 3 (see Supplementary Materials and Methods) are represented by continuous lines. Average values for F_V_/F_M_ or average fluorescence lifetimes are presented ± standard deviation. Each point represents n = 5 (with the exception of panel (b) which corresponds to n = 10). The experiment was replicated twice; data presented corresponds to one replicate. Fitting parameters and statistical analysis are included in Supplementary Material.

To test if photodamage (*in vivo* and *in vitro*) occurs in parallel with the shortening of τ_AV,_ the time courses of the decrease in the maximum efficiency for primary photochemistry of PSII (F_V_/F_M_) and in the fluorescence lifetime were measured. F_V_/F_M_ corresponds to the ratio of variable to maximum chlorophyll fluorescence. It is known^24^ that a decrease in the maximum efficiency for primary photochemistry of PSII, measured as F_V_/F_M_, reflects the magnitude of PSII photodamage if repair is absent. Both experimental model systems were illuminated with 1300 µmol photons m^−2^ s^−1^. Figure 1a,b show that for both illumination wavelengths and samples, 460 and 660 nm, the F_V_/F_M_ decreased with time. The decrease in the photochemical efficiency was fitted using Eq. 2 (see Supplementary Information) and the fit parameters are presented in Table 1 (and Supplementary Figs 1 and 2). Figure 1a,c show the data obtained for illuminated leaves. Both data sets were fitted to a single exponential decay (with rate coefficient k_PI_ and a residual term), whereas changes in the τ_AV_ in Panel c were fitted to a double exponential. The criteria to fit each signal were based on using the best fitting for the obtained results.

**Table 1:** Values of half-lifetime (T_50_) obtained for decreases in PSII efficiency (F_v_/F_m_) and τ_AV_ for 460 and 660 nm illumination in leaves and BBY’s. T_50_ values are presented ± standard error and amplitudes are presented in parenthesis. The first ratio 660/460 reflects the ratio of T_50_ values obtained for F_v_/F_m_ at 660 and 460 nm. The second ratio 660/460 reflects the ratio of T_50_ values obtained for τ_AV_ at 660 and 460 nm.

	λ (nm)	T_50_ (F_V_/F_M_)	Ratio $\frac{{\bf{660}}}{{\bf{460}}}$	T_50_ (τ_AV_)	Ratio $\frac{{\bf{660}}}{{\bf{460}}}$
*Leaves	460	1.8 ± 0.3 (0.6)	1.81	0.9 ± 0.2 (242)	1.72
	660	3.3 ± 1.0 (0.6)		1.55 ± 0.6 (197)	
**PSII-enriched membranes	460	28.8 ± 5.5 (0.5)	0.85	4.5 ± 0.6 (49)	1.09
	660	24.7 ± 5.5 (0.5)		4.9 ± 0.4 (56)	

^*^Units in hours for T_50_.

^**^Units in minutes for T_50_.

In order to facilitate a comparison between different treatments, the time point where 50% of the changes were observed (T_50_ = ln 2/k_PI_) were compared for both experimental model systems. It was observed for illuminated leaves that for 460 nm illumination, T_50_ is smaller than for 660 nm (see Table 1). These data are in good agreement with the previously reported action spectra of photodamage for different species^3,12,21,25^ where the blue region of the visible spectrum induced more damage than the red region. By contrast in leaves the decay of F_V_/F_M_ for both wavelengths was the same in BBY’s (see Table 1).

Photodamage was accompanied by shortening of τ_AV_ in both leaves and BBY’s, as presented in Fig. 1c,d. In the case of leaves (Fig. 1c) illumination with 460 nm light induced a faster shortening of τ_AV_ than illumination with 660 nm light. This shortening of τ_AV_ can be described by a double-exponential decay and the fitting parameters are presented in Table 1 (and Supplementary Table 2).

BBY’s do not show a statistically significant difference in τ_AV_ for 460 nm and 660 nm light (Table 1). The observed time course does not follow a simple first-order rate law; at least two components are needed (Eq. 3, see Supplementary Information). This can be interpreted as two quenching processes occurring in BBY’s. It is remarkable that for both wavelengths most of the changes in τ_AV_ occur during the first minutes of illumination.

It has been hypothesized that PSII photodamage increases the yield of fluorescence in the F_O_ state^24,26^. An increase of F_O_ corresponds to an increase of the average fluorescence lifetime of PSII. However, it was reported^23^ that even in the absence of NPQ, τ_AV_ decreased with illumination time when measured in the F_M_ state (closed PSII RCs). In the dark-adapted state, as measured in present study, the τ_AV_ is expected to be shortest under control conditions since all RCs are expected to be open. Energy cannot be used more efficiently by the RCs than in the dark adapted state. Therefore, the observed shortening of τ_AV_ must be interpreted as an energy dissipation event which is different from primary photochemistry. It is worth noting that under control conditions (darkness across the time course) no significant changes in τ_AV_ and F_V_/F_M_ were observed *in vivo* or *in vitro* (Supplementary Fig. 2).

In Supplementary Fig. 3 (shifting of histograms) and 4 (representative micrographs) the changes in τ_AV_ of leaves after 460 and 660 nm illumination are shown and the values of τ_AV_ are colour coded. The various images taken at different time points show that τ_AV_ shortens during the illumination experiment for both illumination wavelengths. We observed that a change in τ_AV_ occurred in the whole chloroplast and that there was heterogeneity among chloroplasts within the same focal plane.

### The decrease in average fluorescence lifetime is faster than the increase in PSII photodamage

If the changes in τ_AV_ are a consequence of photodamage that leads to the decrease of F_V_/F_M_, they should occur at an equal or lower rate as changes in PSII efficiency. Which is not what is observed in the present experimental observation.

Table 1 shows that τ_AV_ in leaves (0.9 h at 460 nm and 1.6 h at 660 nm) decreased faster than PSII efficiency (1.8 h at 460 nm and 3.3 h at 660 nm) for both wavelengths (T_50_ for decrease in τ_AV_ is 2 times smaller than the T_50_ for photodamage, see Table 1). This implies that the decrease in τ_AV_ cannot be solely explained by loss of maximum PSII efficiency. Similarly, BBY’s showed a faster decrease in τ_AV_ (4.5 min at 460 nm and 4.9 min at 660 nm) than PSII efficiency (∑28 min for both wavelengths); T_50_(τ_AV_) is 3.4 times smaller than T_50_(F_v_/F_m_) in the case of 460 nm excitation, while for 660 nm it is 6.1 times smaller. Despite the differences in complexity between *in vivo* and *in vitro* (BBY’s do not have an unlimited source of electron acceptors, PSI is absent, and other molecular pathways are not present), both experimental model systems showed: (1) photodamage shows single exponential behaviour; (2) higher rate of photodamage induced by 460 nm than by 660 nm light; (3) shortening of τ_AV_; (4) a faster decrease of τ_AV_ than of PSII efficiency. All these observations support the idea that the mechanism(s) of photodamage and quenching *in vitro* and *in vivo* have a common origin.

### Analysis of the fluorescence lifetime components and populations shows that there are at least two types of quenchers formed, one dependent on and one independent of photodamage

**Figure 2:**
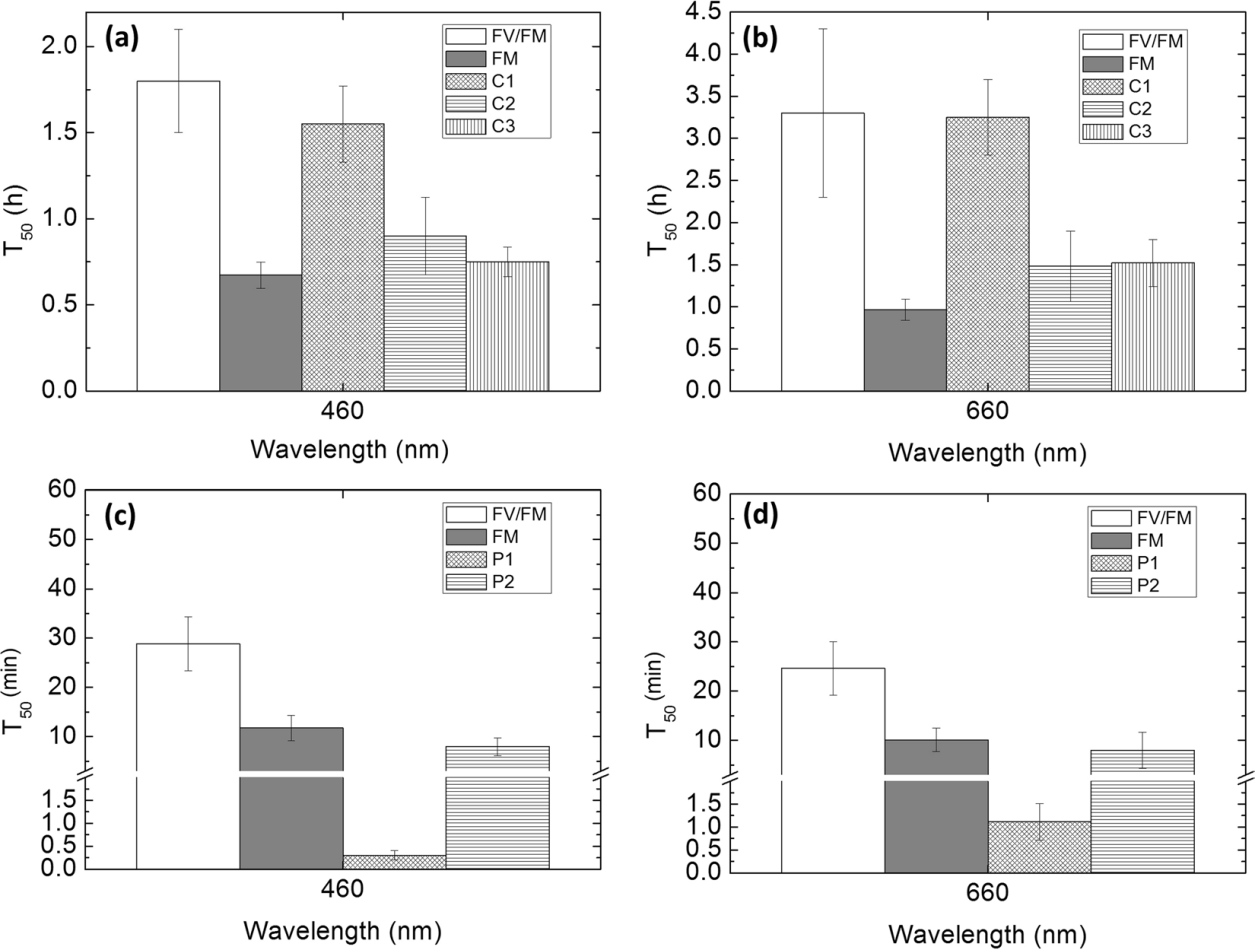
Values of half-time (T_50_) obtained for changes in the individual decay components (a_i_*τ_i_) of fluorescence τ_AV_ of leaves for 460 Panel (a) and 660 Panel (b) nm illumination, and formation of two populations of quenched PSII’s in BBY’s Panel (c) for 460 nm and Panel (d) for 660 nm), compared to the T_50_ values of F_V_/F_M_ and F_M_. T_50_ values are presented with standard error. Each point represents n = 5 (with the exception of the F_V_/F_M_ of BBY’s which corresponds to n = 10). The experiment was replicated twice, and data presented corresponds to one replicate. Fitting parameters and statistical analysis are included in Supplementary Material.

In leaves, decrease in τ_AV_ can be satisfactorily fitted with a single exponential, so only one population of quenched PSII was observed. However, it is possible to analyse the three individual lifetime components of the τ_AV_ (each lifetime component, a_i_*τ_i_, has its own amplitude and lifetime) to establish the relative contributions of individual quenching events to the overall decrease in τ_AV_. It is possible to discern the presence of either one or several quenchers by comparison between the three individual lifetime components of τ_AV_ to T_50_ of loss of F_V_/F_M_ (Fig. 2a,b). It was observed that the first component (C1 = a_1_*τ_1_, which had the shortest fluorescence lifetime) has a T_50_ value similar to that of the loss of F_V_/F_M_) for both illumination wavelengths. The second component of the fluorescence lifetime (C2) had shorter T_50_ values (faster development) than that of photodamage but similar to the T_50_ of the decrease of F_M_ at both wavelengths. The T_50_ of the third component (C3) was statistically similar to that of F_M_ decrease for 460 nm but that was not the case at 660 nm. This suggests that there are at least two distinct quenching processes occurring in parallel in leaves, one where the quencher is formed as a result of photodamage and another where the quencher (represented by C2) is dependent only on illumination. The latter was formed faster and explains why the change in τ_AV_ has shorter T_50_ values than the T_50_ of the loss of F_V_/F_M_. It is worth noting that the decrease of F_O_ was very slow and cannot be satisfactorily fitted appropriately to our single exponential model (See Supplementary Fig. 5).

In *in vitro* measurements, decrease in τ_AV_ can be satisfactorily fitted as double exponential indicating two populations of quenched PSII being formed in response to illumination (decrease of τ_AV_). The first (fast) population was quenched almost immediately after illumination started (0.3 and 1.1 min for 460 and 660 respectably) and the second population formed in a slower manner (approximately 8 min). The decay in F_O_ was remarkably faster and it was not possible to fit the data points in a satisfactory manner (see Supplementary Fig. 5). By contrast, the T_50_ for the changes in F_M_ is statistically similar to the T_50_ of the slow population. None of the two quenched populations seemed to correspond to photodamaged PSII as none of them matched the T_50_ of decrease of PSII maximum efficiency. The analysis of individual components of BBY’s was not possible as each component followed a complex kinetics that did not adjust to first or second order rate laws and for this reason were not taken into account for interpretation.

### Photodamage to the RC is slower than Total PSII inactivation and dependent on excessive pigment excitation

Three independent reports (^3,4,12^) have shown that RC photoinactivation is slower than Total PSII inactivation. Also, it was shown than RC inactivation is dependent on excessive pigment excitation. We have shown that quenchers can be formed before Total PSII inactivation takes place, so it is within reason to hypothesize that the observed quenchers photoprotect the RC to some extent. For this reason, we have estimated the effect on the electron transport rate (ETR) activity of PSII at the time point corresponding to T_50_ of photodamage (which was estimated by PSII efficiency).

**Figure 3:**
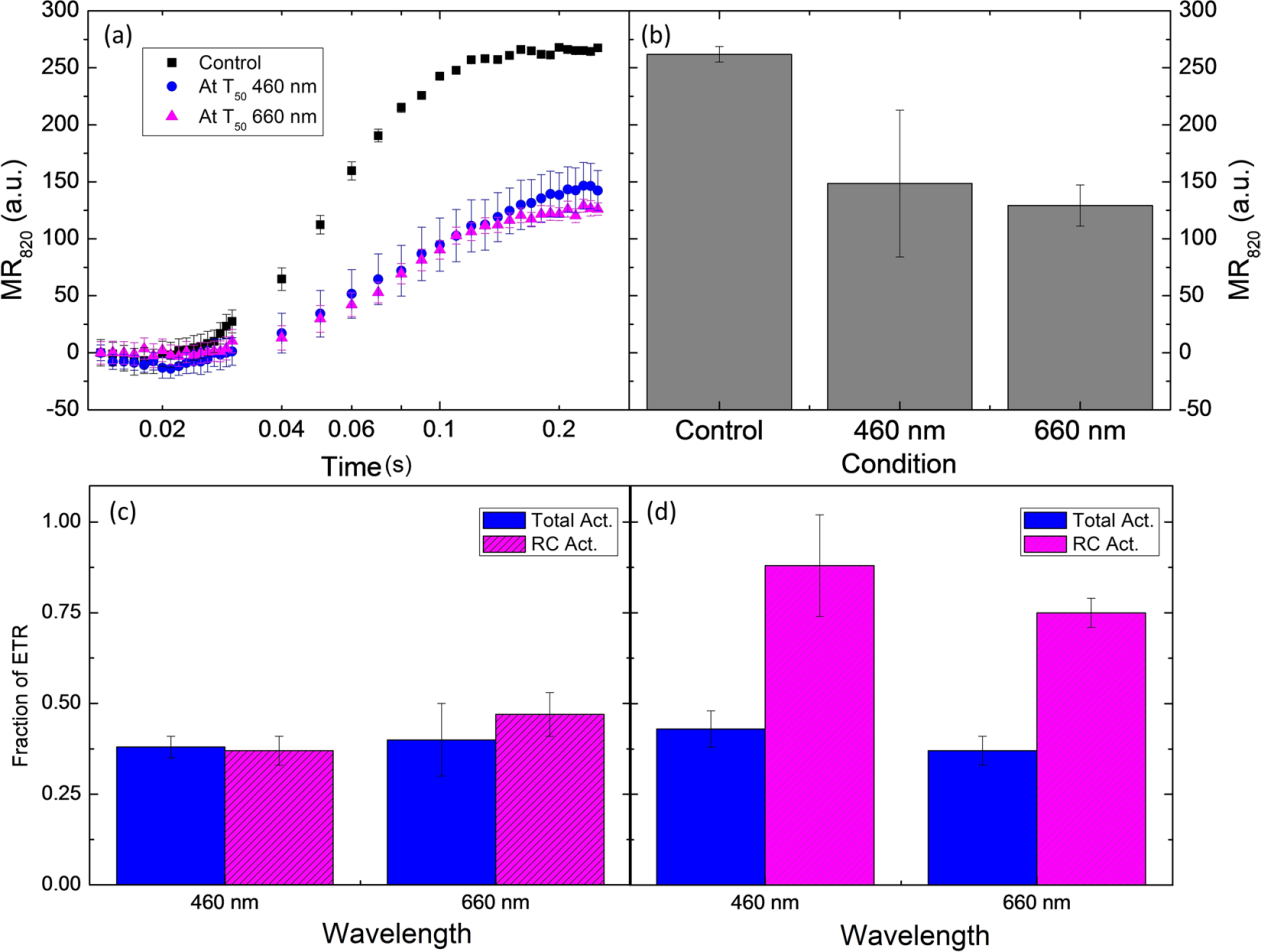
Panel (a): modulated reflectance changes at 820 nm of P700 in leaves for control and both illumination wavelengths (460 and 660 nm) at the T_50_ time point. Panel (b) displays the net amplitude of the P700 accumulation at 220 ms for control and both illumination wavelengths (460 and 660 nm). In panel (c,d) the fraction of ETR of BBY’s is shown the corresponding value of T_50_ of photodamage (min) of illumination. ETR was estimated through absorption changes of DCPIP at 600 nm. Total PSII activity (act.) was calculated from H_2_O to DCPIP and reaction centre (RC) activity (act.) was calculated from DPC to DCPIP. Panel (c), an experiment with no chemicals added during photodamage experiments (control). In panel (d), PPBQ (300 µM) and DPC (300 µM) were present during photodamage experiments. Each point represents n = 5; the experiment was replicated three times, and data presented corresponds to one replicate. T-test was performed between Total PSII activity vs. RC activity, significant difference is denoted by * at a p > 0.05.

Due to the difference between *in vivo* and *in vitro* two approaches were used to estimate the effect on ETR. For leaves the relative ETR was estimated using the modulated reflectance changes at 820 nm of P700 by measuring the amplitude between 0.014 s to the maximum reflectance intensity usually located around 0.2 s (during illumination). In Fig. 3a, a comparison between the efficiency to pump electrons from PSII to PSI (re-reduction of P700^+^) is presented for both wavelengths at the T_50_ time point (2 h for 460 nm and 3 h for 660 nm). As expected from the F_V_/F_M_ kinetics, at T_50_ of both conditions PSII activity dropped by the same magnitude, indicating that the electron flow between both systems (ETR) was impaired. Figure 3b displays the net amplitude of the P700 accumulation at 220 ms (maximum P700 value for the control), for 460 nm a decrease in the activity of ∑60% was observed while for 660 nm the decrease was ∑50%. One should note that the difference between illuminations is not statistically significant, but both are statistically different from the control condition. This confirms that PSII of illuminated leaves were indeed photoinactivated.

In order to estimate ETR *in vitro* a colorimetric DCPIP assay was used (Fig. 3c,d). This method allows the two activities to be probed separately (total PSII activity and RC activity, see methods). Photodamaged samples illuminated in the absence of electron acceptors showed that RC inactivation was significantly lower than the loss of total activity (*t-test*_460nm_ < 0.1 and *t-test*_660nm_ < 0.05) at T_50_. This effect was observed to a greater extent if the sample was exposed under the presence of artificial electron donor (DPC) and acceptor (PPBQ), where RC inactivation was only approx. 20% and significantly lower (*t-test* < 0.001 at both wavelengths). This indicates that RC damage is clearly dependent on excitation pressure and slower than total PSII inactivation.

Comparison of the ETR measurements and PSII efficiency for dark controls and photodamage are presented in Supplementary Fig. 6.

## Discussion

In this work, we address the issue that photodamage is accompanied by a decrease in τ_AV_ in two experimental model systems under excessive excitation. We will analyse four possible scenarios that can explain the observed quenching due to PSII photodamage and shortening of τ_AV_:

1. Quenching represents the first alterations in the PSII functionality (there is no photoprotective effect)^27,28^;
2. Long lasting qE, qZ, and qT^29–32^;
3. Dissipation of excessive light energy from active PSII by neighboring inactivated PSII RCs^23,33^, implying that photodamaged PSII centres have a photoprotective role^23^;
4. Accumulation of a photoproduct, not related to photodamaged PSII itself^34^.

Scenario 1 would imply that the observed quenching effect is a direct consequence of photodamage that inactivates PSII. Here, we demonstrate that the overall quenching in the two model systems occurred faster than the accumulation of PSII photodamage. It has been hypothesized that PSII photodamage should increase the yield of fluorescence in the F_O_ state^26^, but this should be manifested as an increase of τ_AV_ which is contrary to experimental observations in both model systems.

Scenario 2 would link some components of qI with residual of qE, qZ, and qT mechanisms. One possible explanation of the long lasting quenching is a manifestation of qI at the antenna^35^, and the decrease of F_V_/F_M_ is due to long lasting qZ. In both *in vivo* and *in vitro* models, F_M_ decreased faster than F_V_/F_M_*. In vivo* the quencher that is accumulated faster seems to be correlated with F_M_ which in turn reflects the changes in the efficiency of energy dissipation^35^. However, this process should not occur *in vitro*, where the decrease in F_M_ is also faster than F_V_/F_M_. For this reason, component 2 cannot be directly related to a long lasting qZ mechanism.

Also, it has been reported that a non-linear relationship of photoprotection and quenching is possible^22^. However, in this work samples were measured several hours after photodamage, indicating that the observed quenching is a long-lived component of qI. It was demonstrated^23^ that quenching during photodamage occurs regardless of the use of DTT and nigericin in leaves which discards the role of qE, qZ, and qT as the sole explanation of the observed quencher. The alternative is that dark-sustained xanthophyll would be accumulated, but this type of quenching has τ_AV_ longer than F_O_^36^, which is contrary to present observations. Furthermore^37,38^, demonstrated that violaxantin conversion occurs very slowly in leaves not acclimated to chilling temperatures, as in the present study, so the contributions of qZ in this particular case would not explain alone the strong quenching effect. Finally, these types of quenching are not present in BBY’s.

Scenario 3 attributes the origin of the quencher to inactive photodamaged RCs^33^, that concomitantly protect neighbouring active PSII by dissipating excessive excitation. This scenario is dependent on PSII photodamage, as the quenchers are the inactivated PSII. In leaves, a direct relation between photoinactivated PSII and formation of quenchers could be established because of the similarities of T_50_ of decrease in the second component of the fluorescence lifetime. However, this scenario is only applicable *in vivo*.

It should be noted that a photoinactivated RC is not equivalent to a closed RC. While both closed RC and photoinactivated RC cannot reduce Q_A_, a closed RC has τ_AV_ in the range of ns and remains active. In contrast, a photoinactivated RC is chemically modified (due to photodamage), it is inactive and we have demonstrated that its lifetime becomes shorter, rather than longer as it is seen in the closed RC.

Scenario 4 considers a photoproduct generated under high light that acts as a quencher of PSII fluorescence, which is not related to PSII photodamage. This scenario is a good candidate to explain the fast quenching observed *in vivo* and *in vitro*, as the observed decrease of τ_AV_ is not related to the accumulation of photodamaged PSII. It has been suggested that long-lived quenchers can be formed in LHCII complexes, originating from chlorophyll cations or other radicals produced from chlorophyll triplets^33^. As these long-lived quenchers in LHCII are expected to be homogeneously distributed across the PSII population and that LHCII is functionally connected to PSII, they would be able to quench excessive excitations efficiently. Thus, we hypothesize that an unidentified quencher located in the LHCII is a reasonable explanation for the fast quenching component.

Such quencher can be present in leaves and BBY’s as both contain functional LHCII. The difference in the lifetime kinetics of shortening of lifetime between the two model systems is due to the difference in the organizational level of both systems (i.e. ability to change stacking or presence of other possible quenchers *in vivo*). This could be the reason why two quenched PSII populations were observed in BBY’s.

Since damage to the RC^3,4^ and the repair mechanism^15^ is dependent on excitation pressure, the formation of the quenchers before PSII photodamage operates as a pre-emptive photoprotective mechanism that dissipates the accumulated excess energy. This is consistent with the observations that the quencher(s) is (are) accumulated faster (faster shortening of τ_AV_) than photodamaged PSII (loss of F_V_/F_M_), thereby slowing down photoinactivation significantly.

The assays to estimate the electron transport rate (ETR) confirmed that PSII was photoinactivated in both model systems. However, the amount of RC damage can only be estimated *in vitro*. RC damage is greater under limited electron transport at the acceptor and donor side, and vice versa, which indicates that RC is susceptible to damage under excessive light excitation. We hypothesize that PSII photodamage is slower than quencher accumulation because the quencher(s) relieve(s) excessive excitation as soon as it is formed.

In summary, the quenchers photoprotect the RC because its photodamage is dependent on excessive excitation and its damage is slower than the loss of the total PSII activity. We hypothesize that the quenching observed in both experimental model systems has the same nature as both models are photodamaged, both models present a quencher that accumulates faster than the photodamaged PSII and the lifetime of both models is shorter than in F_O_ state. This faster-developing quenching process has two implications: (1) the quencher acts as a “pre-emptive” photoprotection mechanism and (2) quencher formation depends mostly on illumination.

## Materials and Methods

### Plant material and sample preparation

Fresh and intact spinach leaves were purchased at local markets (during the months of June till August, Wageningen, NL). The leaves were stored at 4 °C in a cold room with the petiole submerged in tap water until use. Only plants with high PSII efficiency, determined by measuring the PSII photochemical yield (F_V_/F_M_) were selected for all experiments (see chlorophyll (Chl) *a* fluorescence for PSII efficiency measurements). BBY’s were prepared from fresh market spinach as described (see Supplementary Methods).

### Photodamage *in vivo*

Before illumination, to inhibit photorepair, leaves were infiltrated with 5 mM lincomycin by passive petiole infiltration for 12 hours in order to ensure its uptake by the leaf tissue^23,39^. The irradiance at the surface of the leaf during illumination was 1300 µmol photons m^−2^ s^−1^ of narrow band light (460 ± 10 nm or 660 ± 10 nm). The sample was illuminated for 30, 60, 120, 180 and 300 min. The control group was kept under identical conditions in darkness inside the photoinhibition boxes (see Supplementary Methods). The experiment was repeated three times.

### Photodamage *in vitro*

Samples were illuminated with either 460 or 660 nm light for 1, 2.5, 5, 20 or 60 min (1300 µmol photons m^−2^ s^−1^). BBY’s were then centrifuged at 20,000 × *g*, after which they were re-suspended in standard buffer for cryogenic storage. Aliquots of the sample then were flash frozen in liquid nitrogen and stored at −80 °C until use. The experiment was repeated three times.

### PSII activity measurements

Leaves were dark adapted for at least 15 min at room temperature. Each leaf was measured from petiole to tip in at three different regions. After the measurements, the leaves were transferred back to the LED photoinhibition boxes. The PSII efficiency was measured at room temperature with an M-PEA fluorometer according to Strasser *et al*.^40^ (Hansatech Instruments). The actinic light intensity was 3000 photons m^−2^ s^−1^ for 10 s. All sample manipulation occurred under low light of less than 1 µmol photons m^−2^ s^−1^.

To measure the PSII photochemical efficiency of BBY’s, an aliquot of the sample was defrosted at 4 °C and then diluted with Buffer A and centrifuged at 20,000 × *g*. Then the concentration of BBY’s was adjusted to 150 µg of Chl mL^−1^. PSII efficiency was measured by Chl *a* fluorescence using the same protocol described above. 30 µL of the sample was transferred to wet filter paper before measurement.

Colorimetric measurements for BBY’s were carried down as reported^4^.

Additional PSII activity measurements were done using P700 oxidation kinetics as 820 nm reflectance changes with the method of^41^. P700 signal was recorded simultaneously with the chlorophyll fluorescence using a Multifunctional Plant Efficiency Analyser M-PEA (built by Hansatech Instrument Ltd., King’s Lynn, Norfolk, PE30 4NE, UK). The M-PEA sensor unit is designed to detect 820 ± 25 nm modulated light from an LED and the detector is shielded with band pass filters at 820 ± 20 nm.

### Time correlated single photon counting (TCSPC)

Excitation was carried out by 0.2 ps excitation pulses (412 nm) at a repetition rate of 3.8 MHz. The excitation power was reduced with neutral density filters and the detection rate was kept below 30,000 photons s^−1^. The diameter of the excitation spot was 2 mm, and the excitation laser power was kept at 1–5 µW to keep PSII RCs (RC) in the open state. The sample was kept in a flow cuvette (flowing speed ~2.5 mL s^−1^) connected to a sample reservoir (7.5 mL), kept at 20 °C. The optical path length of the cuvette was 3 mm and the optical density of the sample was 0.1 per cm. Fluorescence was collected at right angle to the excitation beam, under magic angle (54.7°) polarization with a 680 nm interference filter (15 nm bandwidth), as described previously^42^.

The instrument response function (IRF) was obtained from the 6 ps decay of pinacyanol iodide in methanol^43^ (the full width at half maximum of the IRF was 35 ps and a resolution of 2 ps per channel was used). Data analysis was performed with a home-built program^44,45^. For the global analysis the decay lifetime components were kept equal for each run of measurements on the sample exposed to a certain illumination time. The fit quality was judged from the residuals of the fit. Fluorescence decay curves were fitted to a sum of exponentials with the amplitudes *a*_*i*_ and fluorescence decay times *τ*_*i*_, convoluted with the IRF^46^.

Average lifetimes were calculated as:

1 $${\tau }_{AV}=\mathop{\sum }\limits_{i=1}^{N}{a}_{i}\times {\tau }_{i}$$

where $\mathop{\sum }\limits_{i=1}^{N}{a}_{i}=1$, a_i_ is the amplitude of the i-th component and τ_i_ is the lifetime of the i-th component.

### Two-photon fluorescence lifetime imaging microscopy (FLIM)

Time resolved measurements on leaves were performed on a FLIM setup described^47^. Fluorescence was selected using a 680 nm interference filter (13 nm bandwidth) and a 770 nm cut-off filter was used to prevent detection of the excitation beam. A neutral density filter was used in the excitation path to reduce the excitation light intensity. The output of the detector was coupled to a Becker & Hickl single-photon counting module (SPC 830)^48^. The time window was set to 256 channels and fluorescence was recorded for 5 min at a count rate of 10,000 counts per second. The IRF was obtained from decay of pinacyanol iodide in methanol.

SPCImage software from Becker&Hickl was used to process FLIM images. Fluorescence decay curves were fitted to a sum of *N* exponentials, convoluted with the IRF, the lifetime components were fitted individually for each pixel. Average lifetimes were calculated using Eq. 1.

## Supplementary information

Supplementary Information

## Data Availability

The datasets generated during and/or analysed during the current study are available in supplementary data and from the corresponding author on reasonable request.
