# Supplementary material for: Two Quenchers Formed During Photodamage of Phostosystem II and The Role of One Quencher in Preemptive Photoprotection
Source: Sci Rep. 2019 Nov 21;9:17275. doi: 10.1038/s41598-019-53030-7 (PMC6872554; doi:10.1038/s41598-019-53030-7)
Supplement: Supplementary file 1 — Supplementary Information [file 41598_2019_53030_MOESM1_ESM.docx]

**SUPPLEMENTARY INFORMATION FOR:**

**TWO QUENCHERS FORMED DURING PHOTODAMAGE OF PHOSTOSYSTEM II AND THE ROLE OF ONE QUENCHER IN PREEMPTIVE PHOTOPROTECTION**

Alonso Zavafer ^¶^ *, Ievgeniia Iermak ^ǂ §^ ^†^, Mun Hon Cheah  ^ˣ^ ^¶^, Wah Soon Chow ^¶^.

^¶^ Research School of Biology, College of Science, The Australian National University, Canberra ACT 2601, Australia

§ Laboratory of Biophysics, Wageningen University, P.O. Box 8128, 6700 ET, Wageningen, The Netherlands

† BioSolar Cells Project Office, P.O. Box 98, 6700 AB Wageningen, The Netherlands

A.Z and I. I. contributed equally to this work

* Correspondence to alonso.zavaleta@anu.edu.au

ǂ Current affiliation: São Carlos Institute of Physics, University of São Paulo, São Carlos, SP, Brazil

ˣ Current affiliation: Dept. of Chemistry, Uppsala University, Uppsala, Sweden

**Supplementary Information.**

**Supplementary Materials and Methods.**

**PSII enriched membrane handling.** Then the sample was solubilized in a standard buffer (400 mM sucrose, 25 mM MES-NaOH, 15 mM NaCl, 5 mM MgCl_2_, pH 6.5), flash frozen in liquid nitrogen (LN_2_) and stored at −80°C until use. Before light exposure, the sample was unfrozen and resuspended in the standard buffer without sucrose (Buffer A). Then it was centrifuged at 16,000 x g for 5 minutes and resuspended again in Buffer A. The chlorophyll content was measured according to ^1^ and adjusted to 150 µg Chl/mL (this will be referred to as PSII enriched membrane stock solution). The sample was kept in darkness at 4°C at all times unless stated otherwise.

**Photoinhibition boxes.** Light was provided by 16 LED’s of 3 W each bolted into the lid of the aluminum boxes (Supplementary Figure 1A-D). In order to suppress the heat irradiated from the LED array, the box lid was attached to a cooling block (Supplementary Figure 1B-D). Water at 4°C passed through the cooling block, and in this way not only the heat was removed but also stability of the LED sources was ensured (Figure 1F). The temperature of the box lid was 6°C at the plate and at the LED it was 12°C. The sample was kept floating in contact with 4°C water inside the box (Figure 1H).


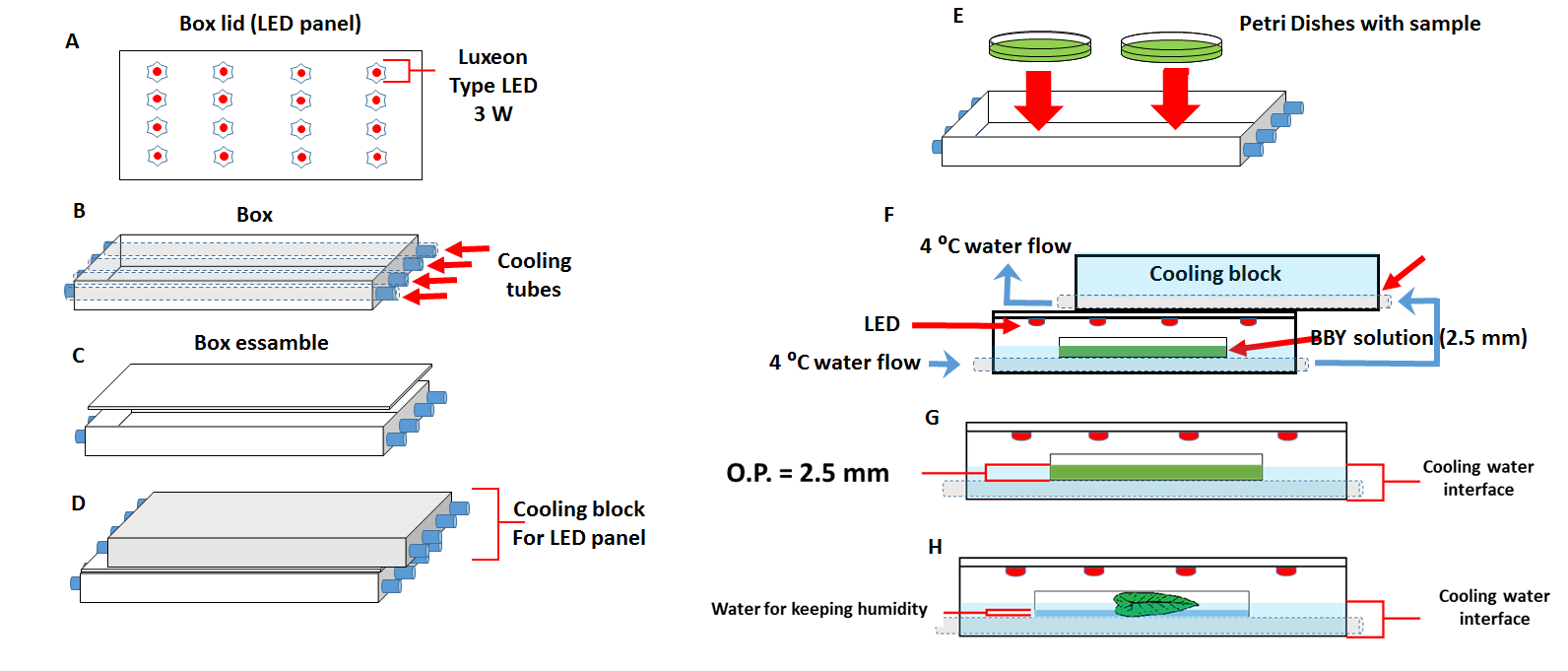


Supplementary Figure 1. Experimental setup used for photodamage experiments *in vivo* and *in vitro*. The box size is 222x146x55 mm.

**Photodamage *in vivo*.** Samples were kept at 4°C at very low light intensity (< 5 µmol photons/m^2^/s) before they were used. PSII activity was measured before illumination using the F_V_/F_M_ Chl a fluorescence parameter ^2^. Leaves were transferred to plastic Petri dishes with a 1 mm layer of water to ensure moistening (Supplementary Figure 1). Each leaf was facing the light on the adaxial side. Then the Petri dishes were placed inside custom made LED light boxes (photoinhibition boxes) made of aluminium (Supplementary Figure 1 A-D).

**Photodamage *in vitro*.** All sample handling for PSII enriched membranes was done at all times at 4°C. The PSII enriched membranes stock solution was diluted with a standard buffer and the concentration of *Chl* was adjusted to 150 µg/mL. Then the sample was transferred to sterile plastic Petri dishes. The optical path length was 2.5 mm. Petri dishes were closed and sealed with Parafilm and transferred to the LED photoinhibition boxes.

**Statistical analysis.** All measured F_V_/F_M_ signals and obtained changes in the fluorescence lifetimes were processed and fitted using OriginPro9.1 software. Changes in PSII activity were fitted, assuming first-order reaction kinetics, to a single-exponential decay function, using Equation (2):

$A_{PSII}\left( t \right)=A_{0}e^{(-k_{PI}\times t)}+y_{0}$ (2)

where *A_PSII_(t)* is the PSII efficiency at a given time *t*, *t* is the time in h/min, *A_0_* is the activity before illumination and *k*_PI_ the rate constant of photoinhibition. Finally, *y_0_* is the residual of the PSII activity.

The changes in the average fluorescence lifetimes for leaves and PSII enriched membranes during photodamage were fitted according to Equation (3):

$\tau_{AV}(t)=\tau_{{AV}_{0}}\times\sum_{i=1}^{n} a_{i}\times e^{{(-k}_{Ti}*t)}+ y_{0}$ (3)

where *τ_AV_(t*) is the average fluorescence lifetime after a given illumination time (see supplementary table 1 and 2 for fitting parameters), τ_AV_*_0_* is the average lifetime of a non-damaged sample, n is the number of decay components (n>1), t is the illumination time (duration of illumination), $a_{i}$ is the amplitude of the i-th component, *k_Ti_* is the corresponding rate constant that describes the change in lifetime of this component, and *y_0_* is the residual of the *τ_AV_*.


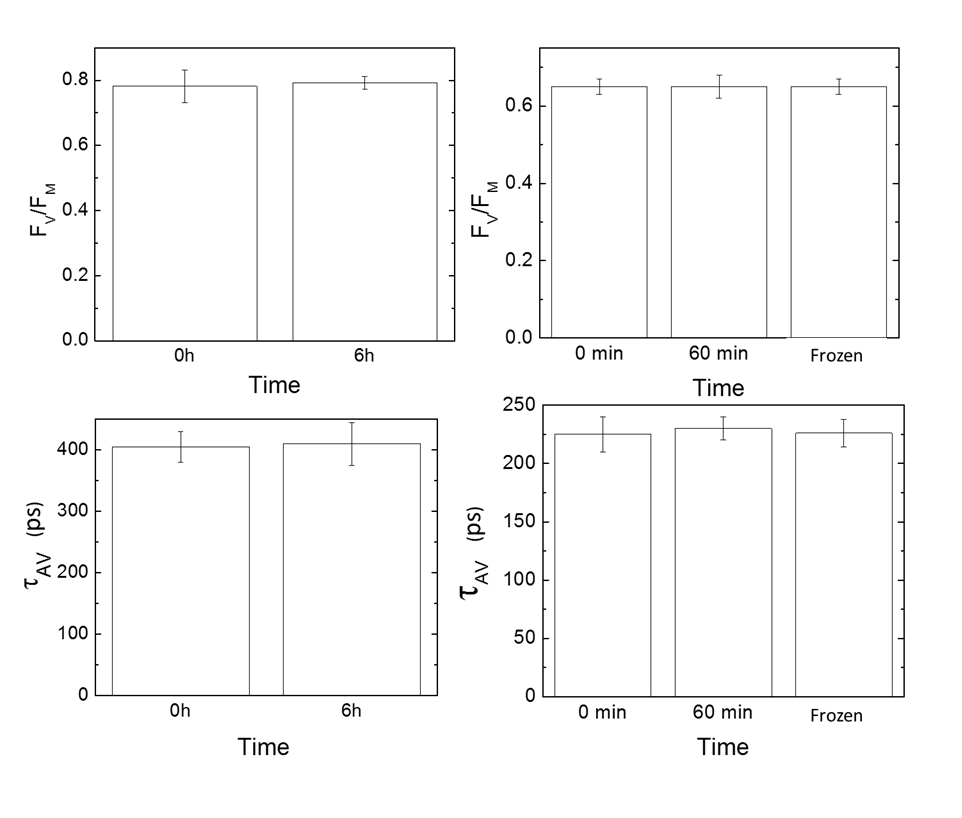


(d)

(c)

(a) (b)

Supplementary Figure 2. Experimental controls (samples kept in the dark). Samples were kept in the exact same manner as the experimental samples. (a) Dark Control Leaves on PSII efficiency during length of the treatment. (b) Dark Control BBY on PSII efficiency during length of the treatment, Frozen refers to effect of re-freeze the sample after the experiment. (c) Dark Control Leaves on τ_AV_ during length of the treatment. (d) Dark Control BBY on τ_AV_ during the treatment, Frozen refer to effect of re-freeze the sample after the experiment. T-test was performed between Total PSII activity vs. RCII activity, significant difference is denoted by * at a p > 0.05.

460 nm 660 nm


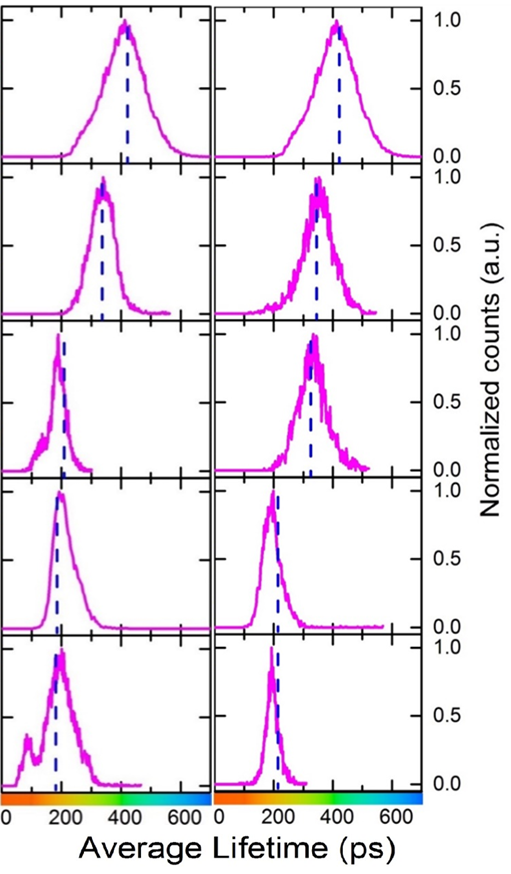


0 h

1 h

2 h

3 h

5 h

6 h

3 h

2 h

1 h

0 h

Supplementary Figure 3. Histograms of the average fluorescence lifetimes of chloroplasts in leaves illuminated with 460 and 660 nm (1300 µmol photons/m^2^/s). Each histogram is the sum of individual histograms for 3-5 replicas. The false colours scale is presented at the bottom. The dotted line represents the mean average lifetime calculated from individual micrographs. Note that the mean average lifetime is not located in the centre of the distribution and does not coincide with the median or the mode which would be expected for a single Gaussian population, indicating that there is more than one population. Histograms are the sum of n = 5 images.

**0 h**

**0 h**


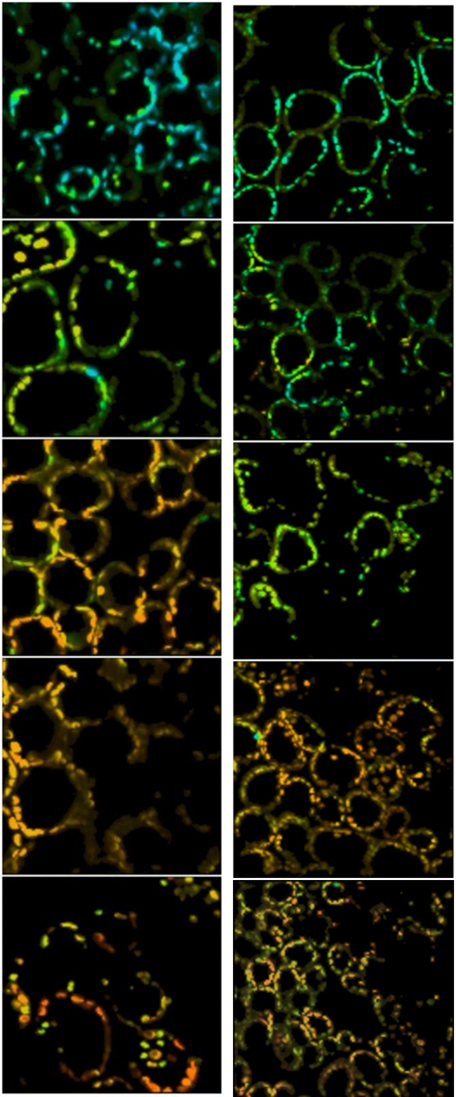

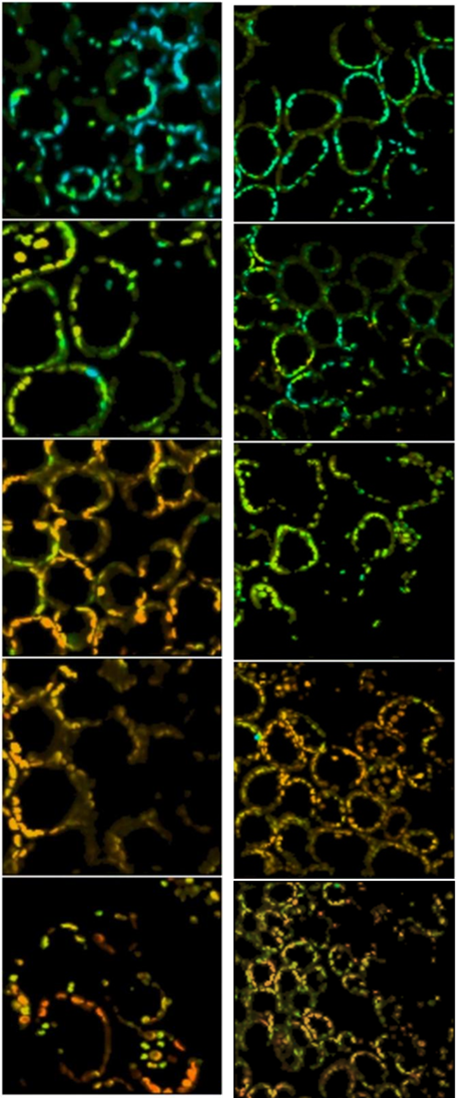


Supplementary Figure 4. Change in the average fluorescence lifetimes of chloroplasts in leaves illuminated with 460 nm light (left column) and 660 nm (right column) (1300 µmol photons m^−2^ s^−1^). Representative FLIM micrographs are shown for each time point (0 h, 1 h, 2 h, 3 h and 6 h of illumination). Micrographs are presented in false colours with the lifetimes ranging from 0 ps (red) to 700 ps (blue). Scale bars = 20 µm. Images were recorded with a 680 nm bandpass filter with a bandwidth of 13 nm.

**1 h**

**1 h**

**2 h**

**3 h**

**6 h**

**5 h**

**3 h**


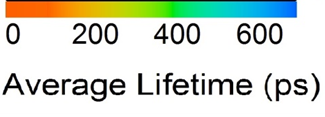


**2 h**


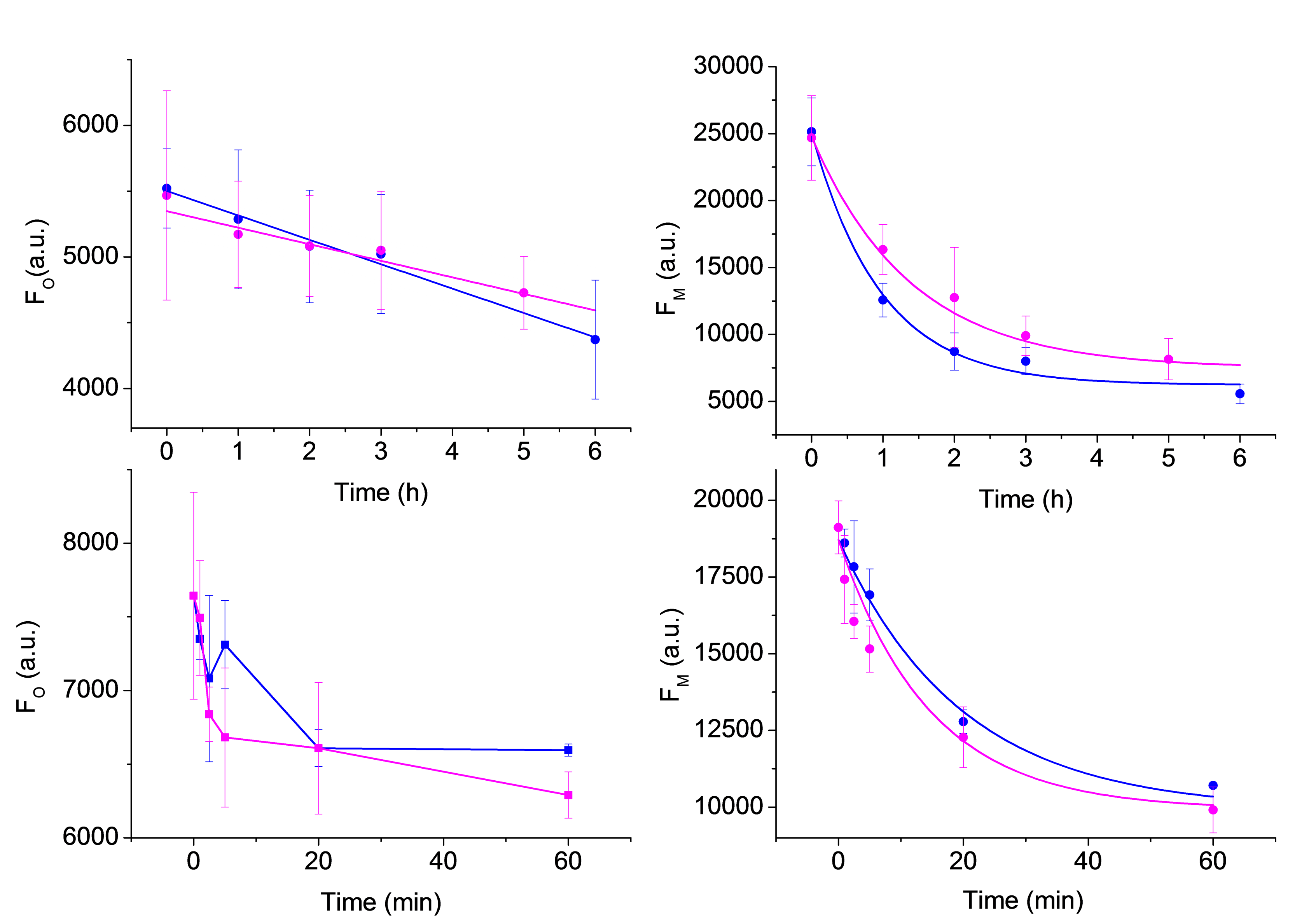


Supplementary Figure 5. Kinetics of the decrease of the fluorescence parameters due to photodamage (blue line for 460 nm illumination and purple for 660 nm) for (a) F_O_ leaves, (b) F_M_ leaves, (c) F_O_ PSII enriched membranes (d) F_M_ PSII enriched membranes. Average values ± std. deviation. Each point represents n $=$5, experiment was replicated twice, data presented corresponds to one replicate.


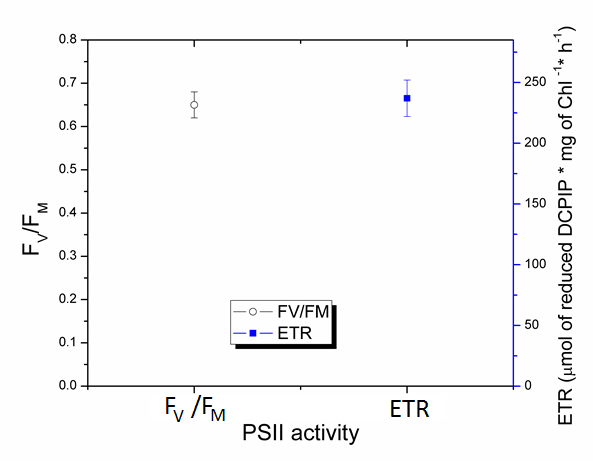


Supplementary Figure 6. Comparison of PSII activity measured by two different means PSII efficiency (F_V_/F_M_) and ETR (colorimetry measured by DCPIP). Each point represents n $=$5.

**Supplementary Tables.**

**Supplementary Table 1.** Parameters for the observed kinetics for the time course of photodamage measured by loss of PSII efficiency (F_v_/F_m_) for 460 and 660 nm illumination. Values are presented ± standard error. The 660/460 reflects the ratio of T_50_ values obtained for F_v_/F_m_ at 660 and 460 nm.

| **PSII photodamage (F_V_/F_M_)** | | | |
| --- | --- | --- | --- |
|  |  | **460 nm** | **660 nm** |
| **Leaves *** | T_50_ | 1.82±0.25 | 3.30±1.04 |
|  | k_PI_ | 3.8E-01±0.5E-01 | 2.1E-01±0.7E-01 |
|  | **Ratio** **T_50_ 660/460** | 1.81 | |
|  | Amplitude | 0.6 | |
| **PSII-enriched membranes**** | T_50_ | 15.43±4.97 | 29.75±8.92 |
|  | k_PI_ | 4.5E-02±1.4E-02 | 2.3E-02±0.7E-02 |
|  | **Ratio** **T_50_ 660/460** | 1.93 | |
|  | Amplitude | 0.5 | |

* Units for k_PI_ and T_50_ in hours

** Units for k_PI_ and T_50_ in minutes

**Supplementary Table 2.** Parameters for the observed kinetics for the change in τ_AV_ for 460 and 660 nm illumination. Values are presented ± standard error. The 660/460 reflects the ratio of T_50_ values obtained at 660 and 460 nm.

| **Change in the τ_AV_** | | | | | | | |
| --- | --- | --- | --- | --- | --- | --- | --- |
| **Leaves *** |  | **460 nm** | | | **660 nm** | | |
|  | T_50_ | 0.90±0.19 | | | 1.55±0.58 | | |
|  | k_T_ | 7.7E-01±1.6E-01 | | | 4.5E-01±1.7E-01 | | |
|  | A | 242 | | | 197 | | |
|  | **Ratio** **T_50_ 660/460** | 1.72 | | | | | |
| **PSII-enriched membranes**** | T_50_^1^ | 0.30±0.10 | k_t1_ | 2.30±1.10 | 1.11±0.4 | k_t1_ | 0.63±0.20 |
|  | T_50_^2^ | 7.97±1.80 | k_t2_ | 0.09±0.02 | 7.97±3.7 | k_t2_ | 0.09±0.04 |
|  | A_1_ | 0.45±0.07 | | | 0.45±0.07 | | |
|  | A_2_ | 0.55±0.06 | | | 0.55±0.06 | | |
|  | Total change in τ_AV_ | 49.00±0.07 | | | 56.00±0.07 | | |
|  | T_50_ | 4.52±0.62 | | | 4.88±0.41 | | |
|  | **Ratio** **T_50_ 660/460** | 1.08 | | | | | |

* Units for k_T_ and T_50_ in hours

** Units for k_T_ and T_50_ in minutes

The partial amplitudes (A_1_ and A_2_) are normalized to the value of total amplitude (A_T_).

**References**

1 Porra, R. J., Thompson, W. A. & Kriedemann, P. E. Determination of Accurate Extinction Coefficients and Simultaneous-Equations for Assaying Chlorophyll-a and Chlorophyll-B Extracted with 4 Different Solvents - Verification of the Concentration of Chlorophyll Standards by Atomic-Absorption Spectroscopy. *BBA-Bioenergetics* **975**, 384-394 (1989).

2 Tyystjarvi, E. & Aro, E. M. The rate constant of photoinhibition, measured in lincomycin-treated leaves, is directly proportional to light intensity. *P. Natl. Acad. Sci. USA* **93**, 2213-2218 (1996).
